# Combined embedding model for MiRNA-disease association prediction

**DOI:** 10.1186/s12859-021-04092-w

**Published:** 2021-03-25

**Authors:** Bailong Liu, Xiaoyan Zhu, Lei Zhang, Zhizheng Liang, Zhengwei Li

**Affiliations:** 1 Engineering Research Center of Mine Digitalization of Ministry of Education, China University of Mining and Technology, Xuzhou, China; 2 School of Computer Science and Technology, China University of Mining and Technology, Xuzhou, China

**Keywords:** MiRNA and disease interactions, Meta-path, Pair embedding, Node embedding, Combined embedding

## Abstract

**Background:**

Cumulative evidence from biological experiments has confirmed that miRNAs have significant roles to diagnose and treat complex diseases. However, traditional medical experiments have limitations in time-consuming and high cost so that they fail to find the unconfirmed miRNA and disease interactions. Thus, discovering potential miRNA-disease associations will make a contribution to the decrease of the pathogenesis of diseases and benefit disease therapy. Although, existing methods using different computational algorithms have favorable performances to search for the potential miRNA-disease interactions. We still need to do some work to improve experimental results.

**Results:**

We present a novel combined embedding model to predict MiRNA-disease associations (CEMDA) in this article. The combined embedding information of miRNA and disease is composed of pair embedding and node embedding. Compared with the previous heterogeneous network methods that are merely node-centric to simply compute the similarity of miRNA and disease, our method fuses pair embedding to pay more attention to capturing the features behind the relative information, which models the fine-grained pairwise relationship better than the previous case when each node only has a single embedding. First, we construct the heterogeneous network from supported miRNA-disease pairs, disease semantic similarity and miRNA functional similarity. Given by the above heterogeneous network, we find all the associated context paths of each confirmed miRNA and disease. Meta-paths are linked by nodes and then input to the gate recurrent unit (GRU) to directly learn more accurate similarity measures between miRNA and disease. Here, the multi-head attention mechanism is used to weight the hidden state of each meta-path, and the similarity information transmission mechanism in a meta-path of miRNA and disease is obtained through multiple network layers. Second, pair embedding of miRNA and disease is fed to the multi-layer perceptron (MLP), which focuses on more important segments in pairwise relationship. Finally, we combine meta-path based node embedding and pair embedding with the cost function to learn and predict miRNA-disease association. The source code and data sets that verify the results of our research are shown at https://github.com/liubailong/CEMDA.

**Conclusions:**

The performance of CEMDA in the leave-one-out cross validation and fivefold cross validation are 93.16% and 92.03%, respectively. It denotes that compared with other methods, CEMDA accomplishes superior performance. Three cases with lung cancers, breast cancers, prostate cancers and pancreatic cancers show that 48,50,50 and 50 out of the top 50 miRNAs, which are confirmed in HDMM V2.0. Thus, this further identifies the feasibility and effectiveness of our method.

**Supplementary Information:**

The online version contains supplementary material available at 10.1186/s12859-021-04092-w.

## Background

Microribonucleic acids (miRNAs), a small non-coding RNA molecule which contains about 21–22 nucleotides, have an important effect on the post-transcriptional level and cell processes [1]. Experiments have confirmed that miRNAs participate in the diagnosis and medical treatment of heart conditions [2], cardiovascular diseases, malignancies, mental disorders and diabetes. For instance, medical experiments exhibit that mir-33 controls cholesterol homeostasis [3]. Hence, it is essential for medical scholars to find out miRNAs which are related to diseases. Many medical technologies, e.g., microarrays and PCR, have been utilized to explore miRNA and disease associations [4]. Though, traditional medical experiments have their limitations in high cost and time-consuming. Therefore, many researchers are devoted to devising computational methods to find unidentified miRNA and disease interactions, so that they can recompense the drawbacks [5, 6] of traditional experimental methods.

Many innovational computational approaches have been developed to discovery miRNA and disease interactions recently. Among them, those methods can be approximately classified into two categories: similarity-based methods and machine learning-based methods. With the presumption that miRNAs with similar functions are closely associated with similar diseases, many kinds of measurements apply similarity-based methods. For instance, Jiang et al. proposed the first method which combines disease phenotype information with miRNA information to predict miRNA and disease interactions [7]. Nevertheless, this approach also had some shortcomings. It was unreasonable to regard the number of overlapping target genes of two miRNAs as the criterion for calculating the miRNA functional similarity score, which proved that it was inadequate because it ignored the indirect neighbors. According to functional similarity, miRNA clusters, and miRNA families, Xuan et al. scored unlabeled miRNAs. However, the miRNA similarity network they utilized restrained their experimental performance [8]. Chen et al. applied the random walk algorithm to the prediction of miRNA and disease associations [9]. However, this method had some limitations in constructing miRNA functionally similar networks, which made it unable to predict new diseases without the confirmed related miRNAs. Then, Chen et al. integrated within-scores and between-scores to rank the unverified miRNA and disease associations [10]. Besides, without using any known miRNA-disease associations, Zhao et al. innovatively constructed a miRNA-lncRNA-disease network(DCSMDA), which integrated the miRNA-lncRNA associations and lncRNA-disease associations to indirectly predict miRNA-disease intearctions [11]. In summary, the subject of the similarity calculation method is to construct a network model, and different methods are used to measure the similarity between nodes in the network to predict miRNA and disease interactions, most of which are limited by the quality of the constructed network model and the incomplete relationship between nodes.

Except methods based on similarity measures, exploring potential miRNA-disease interactions with machine learning algorithms is also a significant academic method in this field. Different from the methods based on similarity to directly calculate the similarity between nodes in the network, researches based on machine learning are committed to extracting inherent features and devising effective classification algorithms to find miRNA and disease associations. For example, Jiang et al. offered negative samples randomly from the unverified miRNA-disease pairs and applied SVM as prediction classifier [12]. Different from above approach, Chen et al. designed a semi-supervised classification, which demanded no negative samples [13]. In order to solve data insufficiency and data noise, Liang et al. devised an objective function based on L1-norm [14]. Chen et al. chose the discriminative features in view of occurrence frequency [15]. Further, Zhao et al. combined multiple weak classifiers with boosting to strengthen classification [16]. In addition, matrix decomposition [17, 18] and collaborative filtering [19] are both useful in revealing miRNA-disease relations. For instance, Mao et al. devised the method based on genomic data fusion, which employed the Bayesian Probabilistic Matrix Factorization model to fuse data from multiple sources(MDBPMF). They innovatively offered a great approximation to the matrix and were able to generalize it by assessing its performance on invisible data [20]. Also, there are enormous efforts on predicting miRNA and disease association motivated by promising development of autoencoder [21], node embedding [22], deep learning and structural deep network embedding (SDNE) [23].

Though, current approaches have favorable performances to predict the unconfirmed miRNA and disease interactions. We still have to do some work to improve experimental performance. On the one hand, many papers have shown that previous node-centric methods simply compute the similarity by applying a similarity metric, such as inner product or Euclidean distance [24], ignoring hidden relative information between two nodes. On the other hand, some methods limit in obtaining intrinsic information and discriminative features from miRNA-disease associations, to a large extent. Moreover, some methods are not suitable for new diseases without the confirmed miRNAs.

**Fig. 1:**
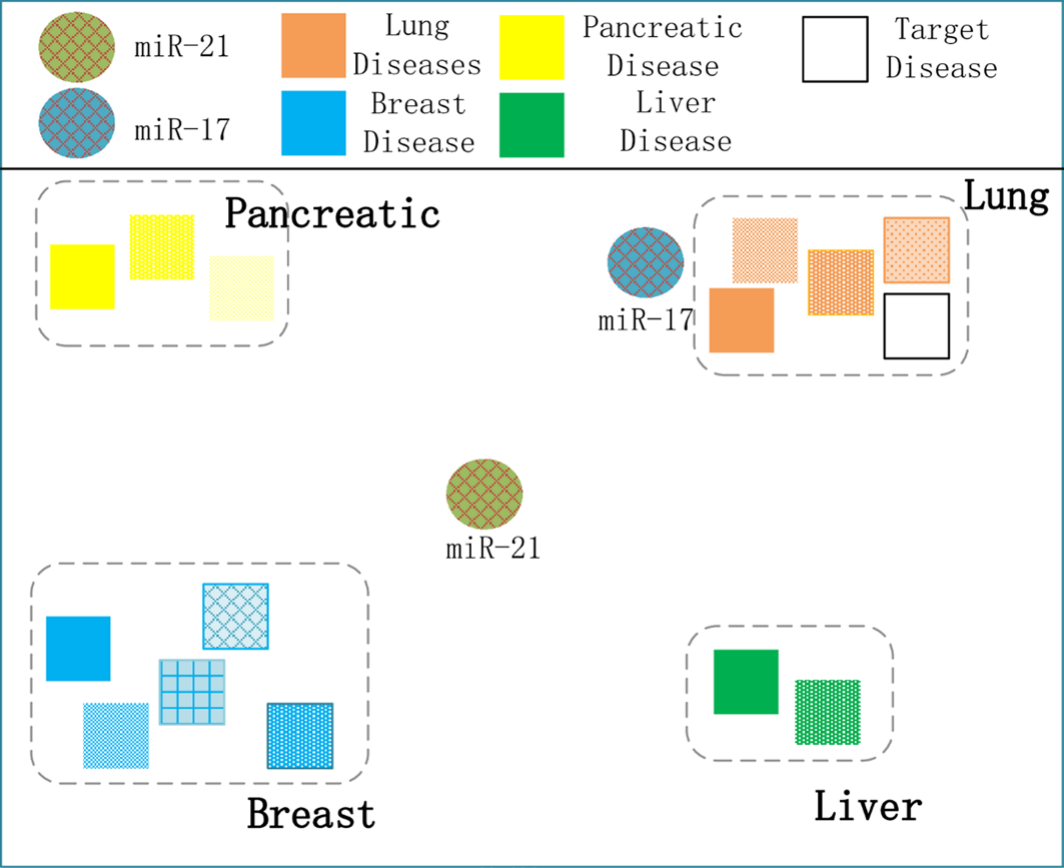
Node embedding

**Fig. 2:**
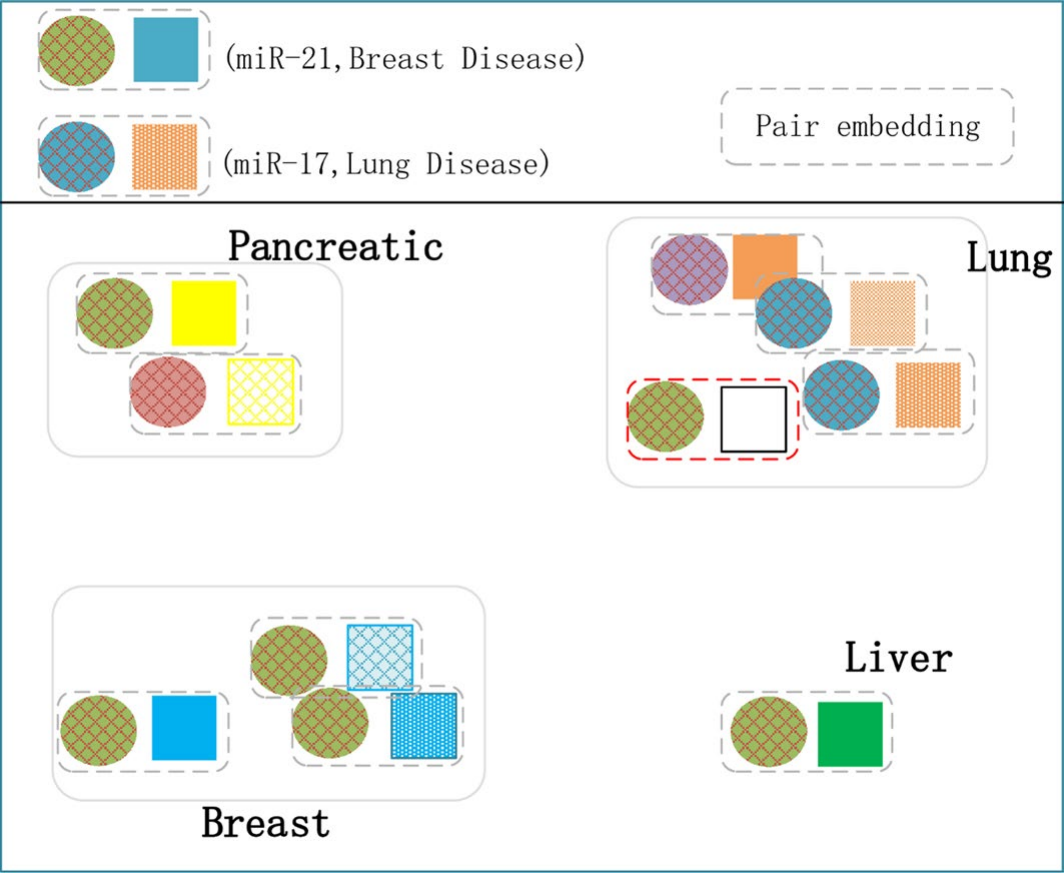
Pair embedding

Node-centric methods fall short of considering the hidden relative information between two nodes. Thence, we introduce the concept of “pair”. We deem that “pair” can better capture the hidden relative features between two nodes. In order to obtain effictient relative features between two nodes, it is necessary to transform the feature them simultaneously which we call “pair embedding”. For instance, Fig. 1 demonstrates a visualization of embeddings of miRNA and disease, where each miRNA is assigned a single embedding. Names of most diseases contain keywords related to body organs, which can be their feature representing their disease type. We assume that miR-21 cluster has related to multiple disease types, such as Pancreatic cancers [25], Breast cancers. Whereas miR-17 cluster [26], regarded as oncogene, is solely overexpressed in lung cancers. Since every miRNA has a single embedding, it has to be embedded to a best single point among all the various disease types. Thus, lung cancers are regarded to be associated with miR-17, rather than miR-21 when predicting. However, in fact, miR-21 has confirmed to be related to lung cancers in clinical trials [27]. On the other hand, as shown in Fig. 2, if we can embed each miRNA-disease pair such that each pair independently captures its associated features. (“Target disease”, miR-21) pair may be associated more closely with the valid pairs related to “lung cancers” than (“Target disease”, miR-17) pair is. To sum up, the pair embedding could capture the hidden features behind the pairwise relationship more precisely than the node embedding.

Meta-paths are some links formed by a series of nodes, which can be employed to preserve associations between nodes and explore the structure information in heterogeneous networks. Shi et al. offered an algorithm to reveal relationships by performing random walk [28]. They used the miRNA-target associations and disease-gene interactions to identify potential miRNA-disease. However, the model strongly depended on the previous nodes to predict the next node in the network [29], ignoring that each node had a different contribution to the meta-path and could not optimize it step by step. Different from Shi’s work, we develop a novel Combined Embedding model for MiRNA and Disease Associations prediction to learn the similarity feature of miRNAs and diseases. We deem that the pair embedding can better capture the features between two nodes. Then, the MLP enables us to construct the fine-grained pairwise relationship in confirmed miRNA and disease pair. We construct heterogeneous network from the identified miRNA-disease pairs, disease semantic similarity and miRNA functional similarity. According to the above heterogeneous network, we find all the associated context paths of each confirmed miRNA and disease in the miRNA-disease heterogeneous network. Then, the associated context paths are linked by nodes, and we propose to employ meta-path based nodding embedding to obtain features which are high contributions to meta-paths during model training. The parameters are optimized to get better prediction through iterative training. To incentivize associated meta-paths, the multi-head attention mechanism is applied to weight the hidden state of each sequence and compensate for the dependency loss of the meth-paths in model training. In this way, the similarity information transmission mechanism in a meta-path of miRNA and disease is obtained through multiple network layers. Finally, we combined the pair embedding and node embedding, which predicts the fine-grained relationship in heterogeneous network better than single embedding. At the same time, CEMDA is suitable for new diseases with unknown miRNA information. Our method outperforms other state-of-the-art methods, with the power of the combination of pair embedding of miRNA-disease and meta-path based node embedding. The results of global LOOCV and 5-folds cross validation illustrate that CEMDA achieves the AUCs of 93.16% and 92.03%, respectively. Furthermore, three kinds of case researches with breast cancers, lung cancers, pancreatic cancers, prostate cancers and colorectal cancers illustrate our approach obtains a remarkable performance.

## Results

Firstly, we present the experimental methods and evaluation criteria. Secondly, compared with five classical methods, the results of CEMDA are analyzed. Finally, we implement three kinds of case researches to verify the experimental performance of our approach.

### Experimental approaches and evaluation criteria

5430 experimental identified miRNAs-diseases interactions are collected from HMDD V2.0 [30] to regard as the dataset in the predicting work. We apply global LOOCV and fivefold cross validation strategies in experiments. Then, every one verified miRNA and disease pair is acted as the testing samples, and the other pairs are view as the training samples in global LOOCV. At the same time, the miRNA and disease associations are divided into five equal-size groups randomly in fivefold cross validation. Then, four groups are regarded as the training set and the other one left acts as the testing set. We repeat fivefold cross validation 50 times to reduce randomness, and then calculate the averaged results. All the meta-paths, the length of which is less than 4, are extracted, because we find that too long meta-paths contribute little to improve the performance and increase too much in computing resources.

We consider area under the curve as AUC, which is regarded as the standard to evaluate the following compared approaches’ performance.

### Comparisons with state-of-the-art methods

**Fig. 3:**
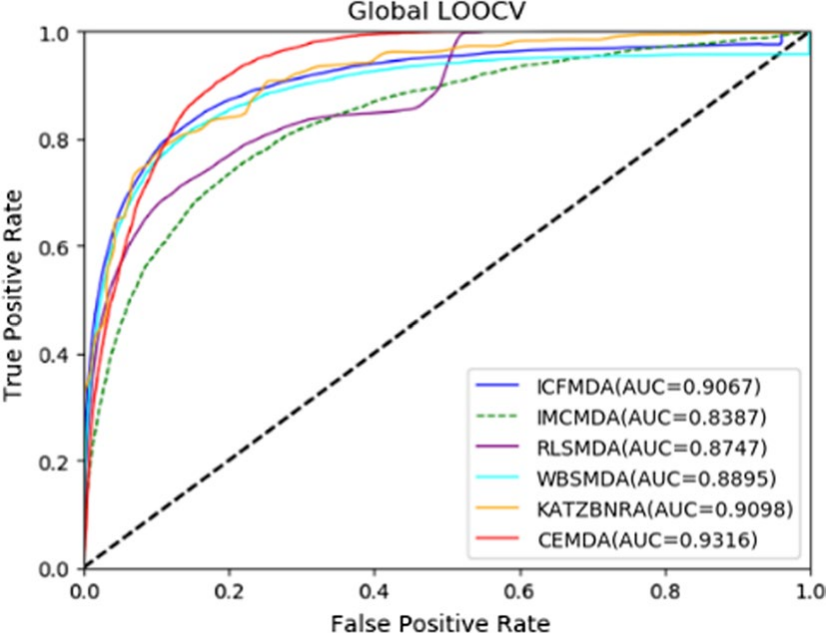
Experimental results of CEMDA, ICFMDA, IMCMDA, WBSMDA, RLSMDA, and KATZBNRA in global LOOCV

**Fig. 4:**
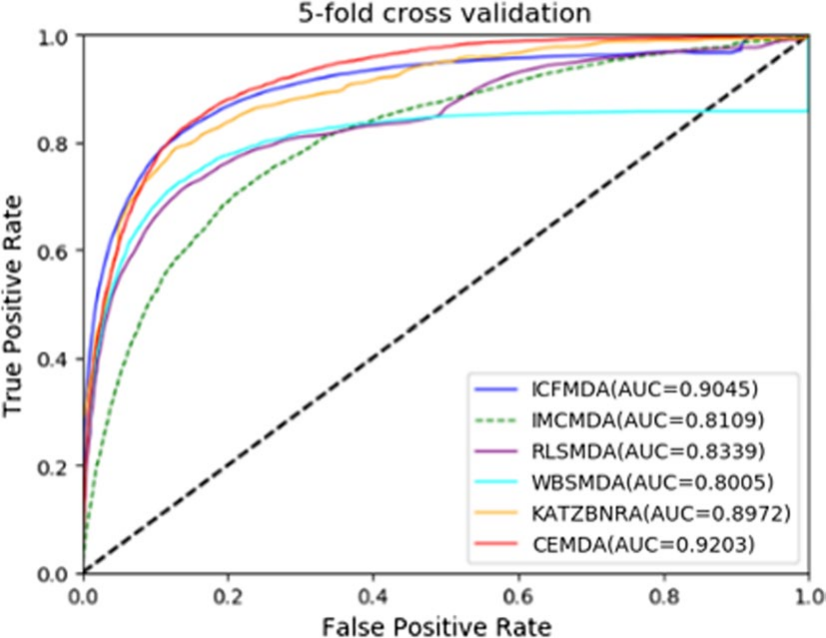
Experimental results of CEMDA, ICFMDA, IMCMDA, WBSMDA, RLSMDA, and KATZBNRA in fivefold cross validation

In order to verify our experimental results, we compared CEMDA with ICFMDA [19], IMCMDA [18], WBSMDA [10], RLSMDA [13] and KATZBNRA [31]. The five compared state-of-the-art approaches in global LOOCV and fivefold cross validation are displayed in Figs. 3 and 4, respectively. Besides, we compare with DCSMDA [11] in global LOOCV and MDBPMF [20] in fivefold cross validation. Since these two methods have only one result, it is not shown in the following figures. As depicted in Fig. 3, CEMDA has the highest AUC of 93.16% in global LOOCV, revealing that it has remarkable performance compared with the other five approaches. Moreover, the AUCs of ICFMDA, IMCMDA, WBSMDA, RLSMDA, KATZBNRA and DCSMDA are 90.67%, 83.87%, 88.95%, 87.47%, 90.98% and 81.55%, respectively. In addition, Fig. 4 shows that CEMDA also achieves the best prediction performance for fivefold cross validation experiments. The AUCs of CEMDA, ICFMDA, IMCMDA, WBSMDA, RLSMDA, KATZBNRA and MDBPMF are, 92.03%, %90.45%, 81.09%, 80.05%, 83.39%, 89.72% and 87.55%, respectively. Therefore, the performance demonstrates that CEMDA is reliable in discovering the unverified miRNA and disease associations.

### Comparisons of CEMDA with pair embedding and without pair embedding

**Fig. 5:**
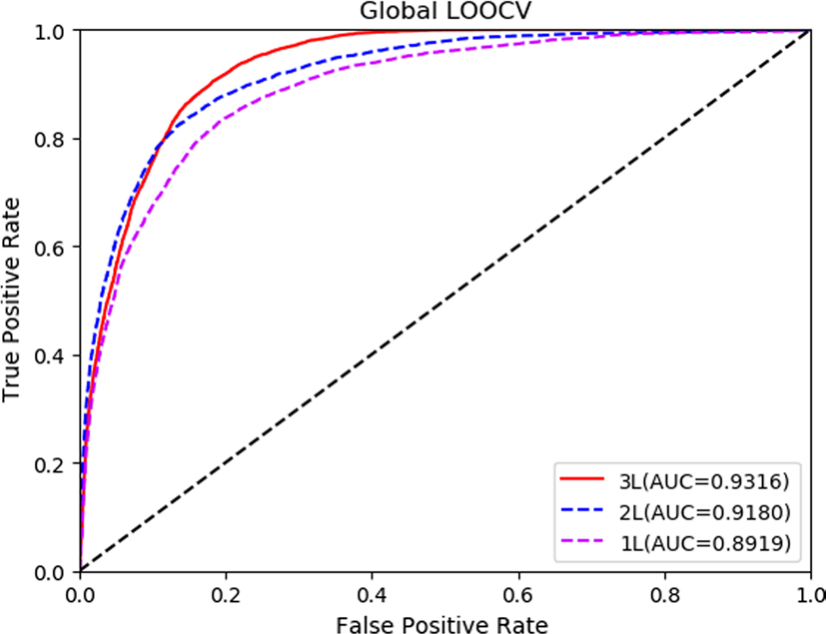
Experimental results of CEMDA with pair embedding and without pair embedding with global LOOCV

**Fig. 6:**
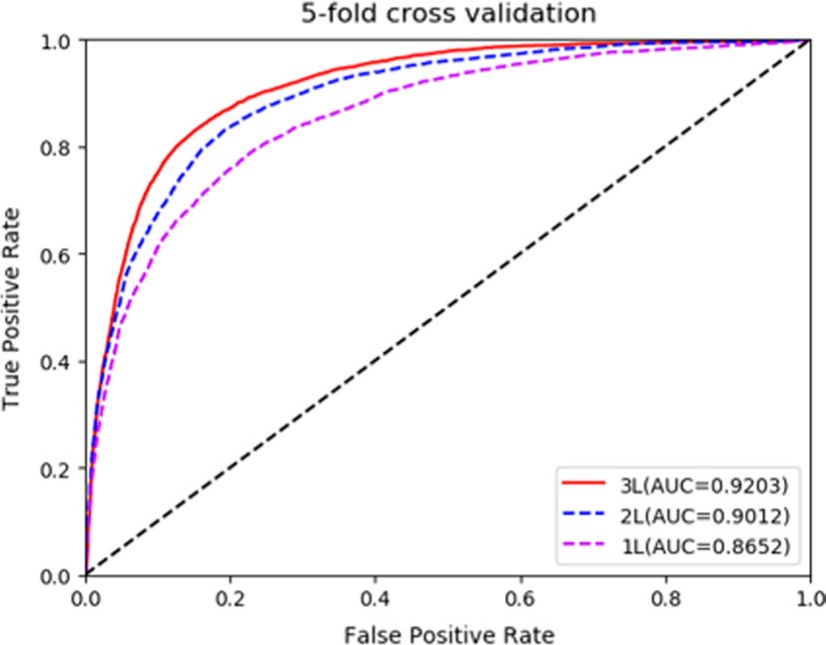
Experimental results of CEMDA with pair embedding and without pair embedding with fivefold cross validation

We compared CEMDA with pair embedding and without pair embedding upon Global LOOCV and fivefold cross validation. The results depicted in Figs. 5 and 6, demonstrate that the pair embedding enhances the effect in global LOOCV and fivefold cross validation strategies, which means that the pair embedding takes an important role in CEMDA. First, the pair embedding helps model the fine-grained pairwise relationship better than the previous when each node only has a single embedding. Second, pair embedding generates incentives to the associated nodes in the meta-path. The feature information of miRNA-disease pair is obtained by multi-layer perceptron to enhance the similarity information transmission.

### Comparisons with different meta-path length of CEMDA

**Fig. 7:**
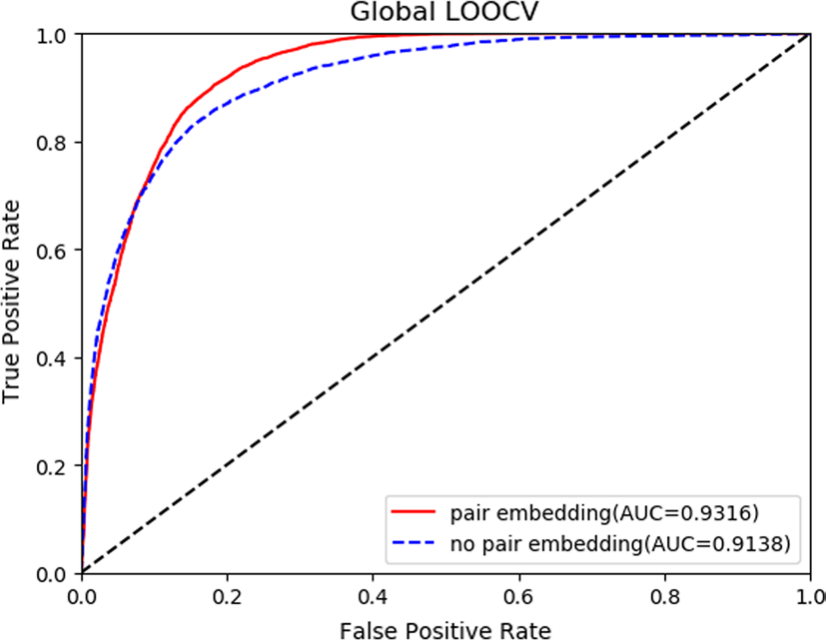
Experimental results of CEMDA with different length of meta-paths in global LOOCV

**Fig. 8:**
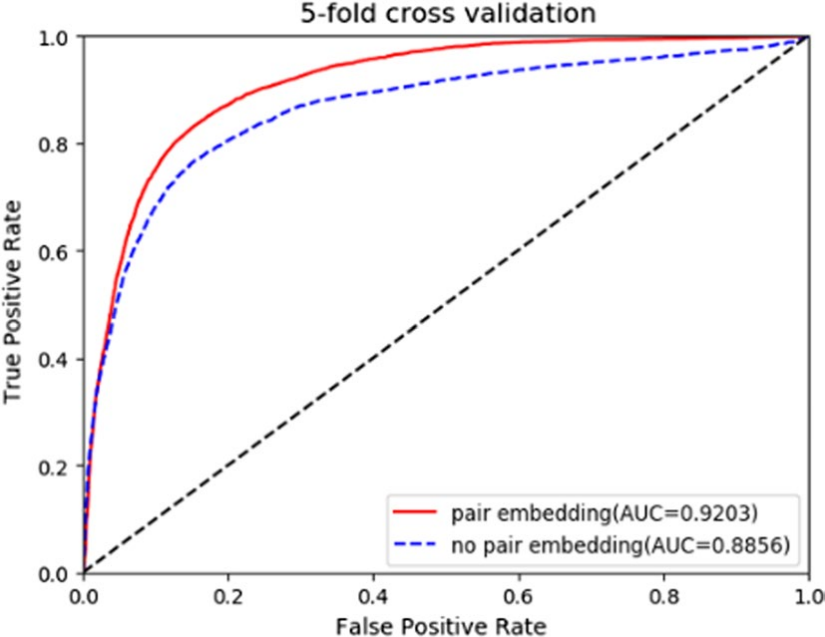
Experimental results of CEMDA with different length of meta-paths in fivefold cross validation

Parameter meta-path length is a critical element for information extraction in CEMDA. Different parameter values result in different information scales. The experimental performance is compared with the different meta-path length upon global LOOCV and fivefold cross validation. Figures 7 and 8 illustrate Experimental results. We find that it’s the better performance when meta-path length increases. More relative nodes are contained when the length of meta-path increases, which brings rich information and abundant features in meta-paths to model training. In other word, the method can integrat more long-term dependency between nodes. Figures 7 and 8 show that the meta-path length increases, but the performance of CEMDA falls distinctly. Because the length of meta-path is longer, the information repeats more in segments that it contains, which contributes less to the performance. After many trials, we decided 3L as the max length of meta-path in our method below.

### Influence of projection dimensions

**Fig. 9:**
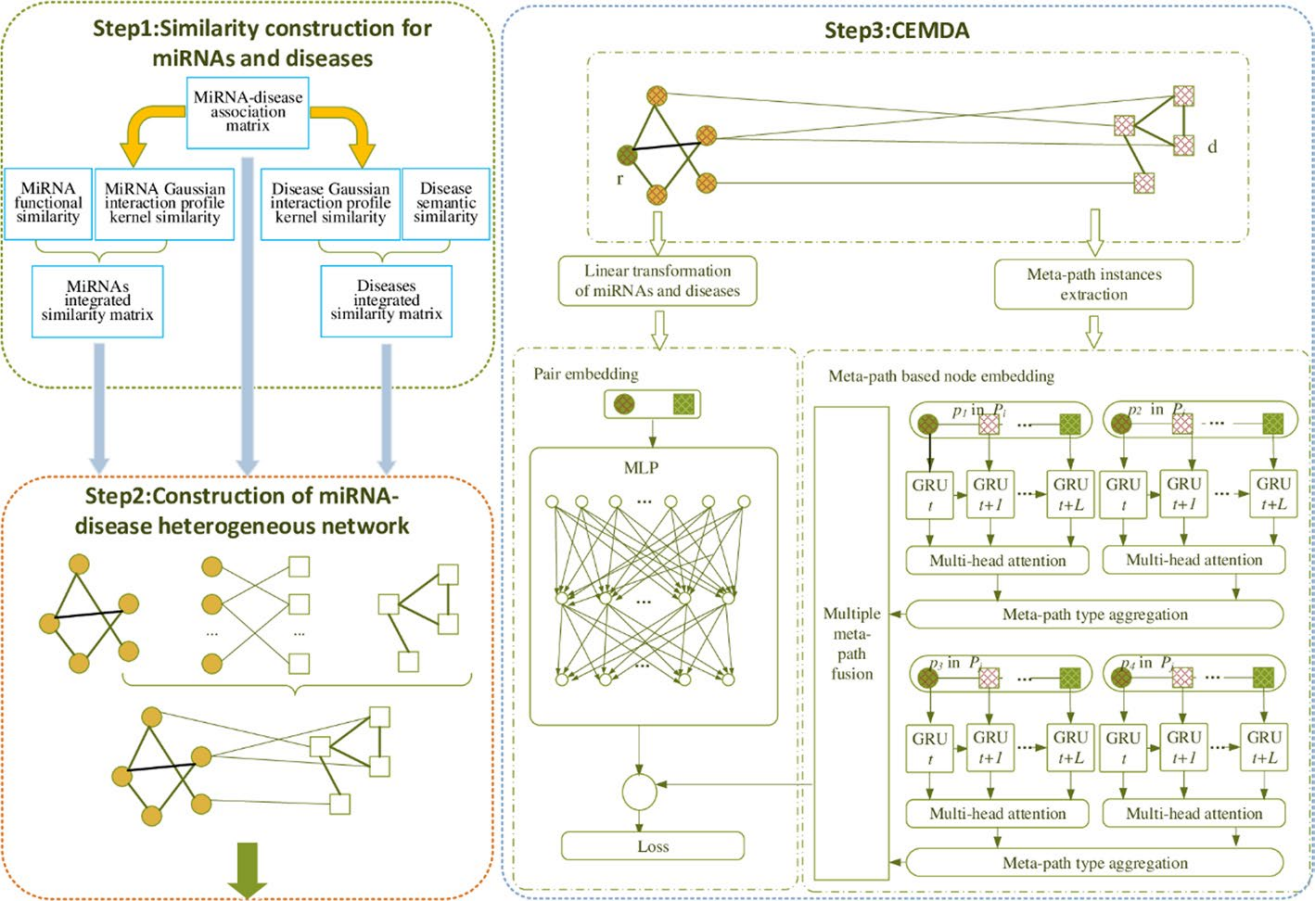
Flow chart of CEMDA

We respectively compared the influence of several projection dimensions $Z$ in Formula (11) on the result of CEMDA under global LOOCV and fivefold cross-validation. Figure 9 shows the AUC values of CEMDA under different projection dimensions $Z$ upon global LOOCV and fivefold cross-validation. In the Formula (11), we used five different projection dimensions, 32, 64, 128, 256 and 512, respectively. It illustrates that the AUC with the increase of projection dimensions values display an upward trend slightly. Besides, we also tested experiment on the projection dimensions of 512, the effect was diminished slightly in training process because of huge amount of calculation and data noise. Thence, we finally selected the projection dimensions of 256.

### Cases studies

Three kinds of case researches are carried out to further validate miRNA and disease interactions. In the first case research, we utilized lung cancers and breast cancers with HDMM V2.0 as data set to discovery the associated unverified miRNAs for. Finally, we compare the found candidate miRNAs with two public databases, dbDEMC [32] and PhenomiR [33] to validate its accuracy.

It has been reported that lung cancers are overwhelming deadly diseases that led to a wide range of deaths worldwide [34]. Biomedical finds that a person discovers lung cancers as soon as possible, he may have a high survival rate. Medical experiments have proven that miRNAs have a huge effect on the diagnosis and cure of lung cancers [35]. Depicted in Table 1, the first column contains the top 50 and the second column lists the top 26–50. Among them, 48 of the top 50 candidates are proved to be related to lung cancers by biological experimental results that are supported from the two public databases. There are only 2 unconfirmed miRNAs. For instance, hsa-mir-421 ranking 1st in the Table 1, has been illustrated to promote proliferation in non-small cell cancers [36]. Thence, the performance of our prediction model offers a novel view for researches.

**Table 1:** The top 50 miRNAs associated with lung cancers

miRNA	Evidence	miRNA	Evidence
hsa-mir-421	dbDEMC, PhenomiR	hsa-mir-92	dbDEMC, PhenomiR
hsa-mir-189	dbDEMC	hsa-mir-105	dbDEMC, PhenomiR
hsa-mir-17	dbDEMC, PhenomiR	hsa-mir-34c	dbDEMC, PhenomiR
hsa-mir-99a	dbDEMC, PhenomiR	hsa-mir-187	dbDEMC, PhenomiR
hsa-mir-20b	dbDEMC, PhenomiR	hsa-mir-149	dbDEMC, PhenomiR
hsa-mir-92	dbDEMC, PhenomiR	hsa-mir-124a	dbDEMC
hsa-mir-302d	dbDEMC, PhenomiR	hsa-mir-320a	dbDEMC, PhenomiR
hsa-mir-28	dbDEMC, PhenomiR	hsa-mir-92b	dbDEMC, PhenomiR
hsa-mir-141	dbDEMC, PhenomiR	hsa-mir-23b	dbDEMC, PhenomiR
hsa-mir-329	dbDEMC	hsa-mir-15a	dbDEMC, PhenomiR
hsa-mir-320e	dbDEMC	hsa-mir-107	dbDEMC, PhenomiR
hsa-mir-378	dbDEMC, PhenomiR	hsa-mir-122	dbDEMC, PhenomiR
hsa-mir-15b	dbDEMC, PhenomiR	hsa-mir-422a	dbDEMC, PhenomiR
hsa-mir-371	dbDEMC, PhenomiR	hsa-mir-377	dbDEMC, PhenomiR
hsa-mir-153	dbDEMC, PhenomiR	hsa-mir-383	dbDEMC, PhenomiR
hsa-mir-663	PhenomiR	hsa-mir-141	dbDEMC
hsa-mir-374b	dbDEMC, PhenomiR	hsa-mir-342	PhenomiR
hsa-mir-584	dbDEMC, PhenomiR	hsa-mir-425	dbDEMC, PhenomiR
hsa-mir-202	dbDEMC, PhenomiR	hsa-mir-377	dbDEMC, PhenomiR
hsa-mir-10a	dbDEMC, PhenomiR	hsa-mir-423	PhenomiR
hsa-mir-16	dbDEMC, PhenomiR	hsa-mir-130b	dbDEMC, PhenomiR
hsa-mir-181d	dbDEMC, PhenomiR	hsa-mir-328	dbDEMC, PhenomiR
hsa-mir-129	dbDEMC, PhenomiR	hsa-mir-515	Unconfirmed
hsa-mir-147b	dbDEMC, PhenomiR	hsa-mir-320d	dbDEMC
hsa-mir-410	PhenomiR	hsa-mir-323b	Unconfirmed

Breast cancers are widespread neoplasms with high mortality in women around the world. The deaths of breast neoplasm will up to three million in the future [37]. Evidence that miR-142-3p is related to breast cancers, has been validated in biological experiments. We adopt CEMDA to verify the related miRNAs for breast cancers and chose the top 50 related miRNAs contained in Table 2. It has been shown that all the top 50 miRNAs were supported by the above-mentioned databases. Hsa-mir-140, which ranks 1st, has been validated to promote the spread of breast neoplasm cell [38]. Thence, the novel findings illustrate that CEMDA offers strong evidence for breast neoplasm predictions.

**Table 2:** The top 50 miRNAs associated with breast cancers

miRNA	Evidence	miRNA	Evidence
hsa-mir-140	dbDEMC, PhenomiR	hsa-mir-125b	dbDEMC, PhenomiR
hsa-mir-18a	dbDEMC, PhenomiR	hsa-mir-611	dbDEMC
hsa-let-7c	dbDEMC, PhenomiR	hsa-mir-372	dbDEMC, PhenomiR
hsa-mir-208a	dbDEMC, PhenomiR	hsa-mir-513c	dbDEMC
hsa-mir-525	PhenomiR	hsa-mir-181d	dbDEMC, PhenomiR
hsa-mir-369	dbDEMC, PhenomiR	hsa-mir-15b	dbDEMC, PhenomiR
hsa-mir-95	dbDEMC, PhenomiR	hsa-mir-32	dbDEMC, PhenomiR
hsa-mir-15b	dbDEMC, PhenomiR	hsa-mir-500a	dbDEMC
hsa-mir-181c	dbDEMC, PhenomiR	hsa-mir-382	dbDEMC, PhenomiR
hsa-mir-302e	dbDEMC	hsa-mir-455	PhenomiR
hsa-mir-329	dbDEMC, PhenomiR	hsa-mir-224	dbDEMC
hsa-mir-337	dbDEMC, PhenomiR	hsa-mir-361	PhenomiR
hsa-mir-30a	dbDEMC, PhenomiR	hsa-mir-520b	dbDEMC, PhenomiR
hsa-mir-186	dbDEMC,PhenomiR	hsa-mir-663	dbDEMC, PhenomiR
hsa-mir-33a	dbDEMC, PhenomiR	hsa-mir-659	dbDEMC
hsa-mir-28	dbDEMC, PhenomiR	hsa-mir-451	dbDEMC, PhenomiR
hsa-let-7f	dbDEMC, PhenomiR	hsa-mir-135	dbDEMC, PhenomiR
hsa-mir-16	dbDEMC, PhenomiR	hsa-mir-193b	dbDEMC, PhenomiR
hsa-mir-330	dbDEMC, PhenomiR	hsa-mir-222	dbDEMC, PhenomiR
hsa-mir-346	dbDEMC, PhenomiR	hsa-mir-199b	dbDEMC, PhenomiR
hsa-mir-371	dbDEMC,PhenomiR	hsa-mir-101	dbDEMC, PhenomiR
hsa-mir-451	dbDEMC, PhenomiR	hsa-mir-510	dbDEMC
hsa-mir-484	dbDEMC, PhenomiR	hsa-mir-105	dbDEMC, PhenomiR
hsa-mir-492	dbDEMC	hsa-mir-183	dbDEMC, PhenomiR
hsa-mir-504	dbDEMC	hsa-mir-33b	dbDEMC, PhenomiR

Then, in the second case research, we want to verify whether this approach is suitable for new diseases without the confirmed related miRNA in biological experiments. We first selected prostate cancers because it is the most universal cancers in men in the world. It is said that over one hundred thousand men die from prostate diseases in a foreign country in 2018 [39]. Firstly, we set all miRNA-disease associations that are associated with prostate cancers from HMDD 2.0 to zero and then perform CEMDA to verify the related miRNAs for prostate cancers. The results shown in Additional file 1: Table S1 indicates that all the top 50 miRNAs were verified by dbDEMC and PhenomiR. Second, to access more new diseases further, we carried out the research on pancreatic cancers. The results of the case of pancreatic cancers are contained in Additional file 1: Table S2. All of the top 50 predicted miRNAs were also included in HMDD, dbDEMC and PhenomiR. Therefore, the case indicates that CEMDA is suitable for new diseases without the confirmed related miRNAs.

Finally, we implemented the third case research to identify whether CEMDA trained with data from an older version of HMMD could verify new imported miRNA and disease pairs in a new version of HMDD. We use HMDD 3.0 [40], dbDEMC and PhenomiR to identify the outcomes. The findings of the case research in colorectal cancers are contained in Additional file 1: Table S3. All of the top 50 miRNAs are supported by HMDD 3.0, dbDEMC and PhenomiR.

In view of the outcomes of three case researches, we summarize that, our approach is effective when predicting unverified miRNA and disease interactions.

## Discussion

Compared with five classical approaches upon global LOOCV and fivefold cross validation, experimental results indicate that CEMDA has better prediction performance. Moreover, three kinds of case researches with five diseases also support our approach’ s result. Firstly, we take out all meta-path instances of the confirmed miRNA and disease pair in miRNA and disease heterogeneous network to obtain complicated associations from miRNA and disease interactions. Meta-paths are linked by noeds and then input to GRU to learn more accurate similarity measures between miRNA and disease. Considering that there are different nodes with different contribution values in the meta path, the multi-head attention mechanism is used to weight the hidden state of each mate-path, and the similarity information transmission mechanism in a meta-path of miRNA and disease is obtained through multiple network layers. Second, the MLP is utilized to obtain the relative information in confirmed miRNA and disease pair. By applying pair embedding that captures the features behind the pairwise relationships, we can obtain the fine-grained associations. Finally, meta-path based node embedding and pair embedding are devised to integrate node and edge information from meta-path instances. In conclusion, CEMDA achieves an excellent prediction in modeling the fine-grained pairwise relationship and considering contributions of different nodes in the miRNA and disease heterogeneous network.

## Methods

The framework of predicting miRNA and disease associations by CEMDA is presented in Fig. 9. Firstly, many similarity methods are utilized to compute miRNA integrated similarity and disease integrated similarity. Secondly, we build the heterogeneous network from experimentally certified miRNA and disease associations, miRNA integrated similarity and disease integrated similarity. Thirdly, we develop a novel Combined Embedding model to extract associated information to predict the unidentified miRNA and disease associations. The model is composed of pair embedding of miRNA-disease, meta-path based node embedding and predicting miRNA-disease associations with combined embedding. Pair embedding employs the MLP to pay more attention to important segments in pairwise relationship. Then, the initial representations of miRNAs and diseases with different dimensions are projected into the same vector space. The associated context paths are serialized based on nodes, and then GRU is used to learn node features which are high contributions to meta-paths. The multi-head attention mechanism is used to weight the hidden state of each sequence, and the entire meta-path information is obtained through multiple network layers. We define the loss function to obtain the ultimate representations of miRNAs and diseases by combining pair embedding and meta-path based node embedding.

### Structure of MiRNA and disease heterogeneous network

#### MiRNA and disease association network structure

HMDD V2.0 is composed of supported experimentally miRNA-disease interactions, which is a universal database. In this article, we employ the adjacency matrix $A\in {R}^{m\times n}$ to express the supported miRNA and disease associations. Where, $m$ and $n$ stand for the number of miRNAs and diseases, respectively. The element ${A}_{ij}$ is equal to 1, which means miRNA ${r}_{i}$ is associated with disease ${d}_{j}$. Otherwise, ${A}_{ij}$ equals to 0 in the matrix. We utilize the datasets with HMDD v2.0 to construct the matrix. As illustrated in the datasets, there are 5430 associations between 495 miRNAs and 383 diseases. We define that $m=495$ and $n=383$. Overall, the adjacency matrix $A$ is adopted to construct miRNA and disease association network.

#### Disease integrated similarity network construction

In order to make the experimental model more accurate and reliable, we investigated Wang et al.’s work [41] and then utilized Medical Subject Headings (MeSH) [42] to calculate the semantic similarity of diseases. We calculate disease integrated similarity network $SD$ by aggregated disease semantic similarity $SS$ and disease Gaussian interaction profile kernel similarity $GD$ as follows:

1 $$SD\left( {d_{i} ,d_{j} } \right) = \left\{ {\begin{array}{*{20}l} {SS\left( {d_{i} ,d_{j} } \right)} \hfill & { d_{i} \;and\;d_{j} \;has\;combined\;sematic\;similarity} \hfill \\ {GD\left( {d_{i} ,d_{j} } \right)} \hfill & {otherwise} \hfill \\ \end{array} } \right.$$

where $GD\left({d}_{i},{d}_{j}\right)$ represents disease Gaussian interaction profile kernel similarity.

Assuming that if two diseases have more the same ancestor subject headings, they will be more similar in semantics. In the above Formula (1), $SS\left({d}_{i},{d}_{j}\right)$ represents the combined semantic similarity of diseases ${d}_{i}$ and ${d}_{j}$. For the first disease semantic similarity method, we take disease semantic similarity based on MeSH which defined by Wang et al. For any kind of disease $D$, it can be represented by a Directed Acyclic Graph $(DAG\left(D\right))$, which contains the set of ancestor disease nodes and the edges of each parent node pointing to the child node. They define the contribution of disease $d$ in $DAG(D)$ as follows:

2 $$D1_{D} \left( d \right) = \left\{ {\begin{array}{*{20}l} 1 \hfill & {if\;d = D} \hfill \\ {max\{ \Delta *D1_{D} \left( {d^{\prime}} \right)|d^{\prime} \in children of d\} } \hfill & {if\;d \ne D} \hfill \\ \end{array} } \right.$$

where $\Delta$ is the semantic attenuation contribution factor (0 < ∆ < 1). This article refers to Xuan et al.’s study [8] and set factor $\Delta$ to 0.5. Then, the semantic value of disease $D$ is the sum of the semantic contribution values of $D$ and its all ancestor nodes as follows:

3 $$DV1\left( D \right) = \mathop \sum \limits_{d \in T\left( D \right)} D1_{D} \left( d \right)$$

where $T(D)$ means all ancestor nodes of disease $D$ including itself in the $DAG$ graph.

Eventually, they calculate the first disease semantic similarity between disease ${d}_{i}$ and disease ${d}_{j}$ as follows:

4 $$SS1\left( {d_{i} ,d_{j} } \right) = \frac{{\mathop \sum \nolimits_{{d \in T\left( {d_{i} } \right) \cap T\left( {d_{j} } \right)}} \left( {D1_{{d_{i} }} \left( d \right) + D1_{{d_{j} }} \left( d \right)} \right)}}{{DV1\left( {d_{i} } \right) + DV1\left( {d_{j} } \right)}}$$

Xuan et al. [8] defined the second method to provide the semantic value of disease $D$. Supposing that some special diseases may have higher contributions to disease $D$, they have another definition of the semantic contribution of disease $d$ as follows:

5 $$D2_{D} \left( d \right) = - log\frac{{the\;number\;of\;DAGs\;inluding\;d{ }}}{the\;numbuer\;of\;diseases}$$

When, the semantic similarity $SS2\left({d}_{i},{d}_{j}\right)$ between ${d}_{i}$ and ${d}_{j}$ is calculated as the percentage of the contribution of themselves and their common ancestor nodes as follows:

6 $$SS2\left( {d_{i} ,d_{j} } \right) = \frac{{\mathop \sum \nolimits_{{d \in T\left( {d_{i} } \right) \cap T\left( {d_{j} } \right)}} \left( {D2_{{d_{i} }} \left( d \right) + D2_{{d_{j} }} \left( d \right)} \right)}}{{DV2\left( {d_{i} } \right) + DV2\left( {d_{j} } \right)}}$$

Eventually, the first disease semantic similarity calculation method and the second disease semantic similarity calculation method are arithmetically averaged as the disease semantic similarity $SS\left({d}_{i},{d}_{j}\right)$ as follows:

7 $$SS\left( {d_{i} ,d_{j} } \right) = \frac{{{ }SS1\left( {d_{i} ,d_{j} } \right) + { }SS2\left( {d_{i} ,d_{j} } \right)}}{2}$$

Finally, according to the Formula(1), we calculated disease integrated similarity network $SD\left({d}_{i},{d}_{j}\right)$.

#### MiRNA integrated similarity network structure

According to Wang et al.’ study, miRNAs with similar functions are often associated with diseases with similar semantics [42]. We calculated miRNA similarity by merging miRNA functional similarity $FS$and Gaussian interaction profile kernel similarity $GM$ as follows:

8 $$SM\left( {r_{i} ,r_{j} } \right) = \left\{ {\begin{array}{*{20}l} {{ }FS\left( {r_{i} ,r_{j} } \right)} \hfill & {r_{i} \;and\;r_{j} \;has\;functional\;similarity} \hfill \\ {GM\left( {r_{i} ,r_{j} } \right)} \hfill & {otherwise} \hfill \\ \end{array} } \right.$$

where $FS\left({r}_{i},{r}_{j}\right)$ ($i\in \left[{1,495}\right], j\in [{1,383}])$ represents miRNA functional similarity between ${r}_{i}$ and *r*_*j*_. *GM*(*r*_*i*_, *r*_*j*_) represents Gaussian interaction profile kernel similarity of miRNAs ${r}_{i}$ and ${r}_{j}$. Benefit from Wang’s task, the miRNA functional similarity $FS\left({r}_{i},{r}_{j}\right)$ is downloaded from their study.

Besides, Zhao et al. calculated the Gaussian similarity calculation between miRNA ${r}_{i}$ and miRNA ${r}_{j}$ as follows [16]:

9 $$GM\left( {r_{i} ,r_{j} } \right) = \exp \left( { - \alpha_{r} IV\left( {r_{i} } \right) - IV\left( {r_{j} } \right)^{2} } \right)$$

where $IV({r}_{i})$, $IV\left({r}_{i}\right)$ is the *i*-th and *j*-th row of matrix $A$, respectively. Parameter ${\alpha }_{r}$ controls the kernel bandwidth as follows:

10 $$\alpha_{r} = \frac{{\alpha_{r0} }}{{\frac{1}{m}\mathop \sum \nolimits_{i = 1}^{m} IV\left( {r_{i} } \right)^{2} }}$$

where initial kernel bandwidth parameter ${\alpha }_{r0}$ is set to 1.

Finally, we can provide miRNA integrated similarity network $SM$ as Formula (8).

To sum up, we combine miRNA and disease association network, miRNA integrated similarity network, disease integrated similarity network to construct miRNA and disease heterogeneous network. We define MiRNA and Disease heterogeneous network as an undirected graph *G* = ( *V, E*), including miRNAs ($M$) and diseases ($D$). *V* is composed of miRNA and disease nodes. *E* represents an edge set containing three edge types, for example, $M\to D$ or $D\to M$ indicates a miRNA is correlated with a disease, $M\to M$ suggests two miRNA nodes are similar and $D\to D$ reveals us there is an edge between two disease nodes.

### Meta-path instances extraction from MiRNA and disease heterogeneous network

There are one or multiple paths between a miRNA and a related disease in miRNA and disease heterogeneous network. Meta-paths mean that the indirect and composite connections between miRNA and disease, which help to understand information and complicated structure in miRNA and disease associations. There are different meta-path instances between the confirmed miRNA and disease association in its sequence. For convenience, we explain meta-path instance below.

**Fig. 10:**
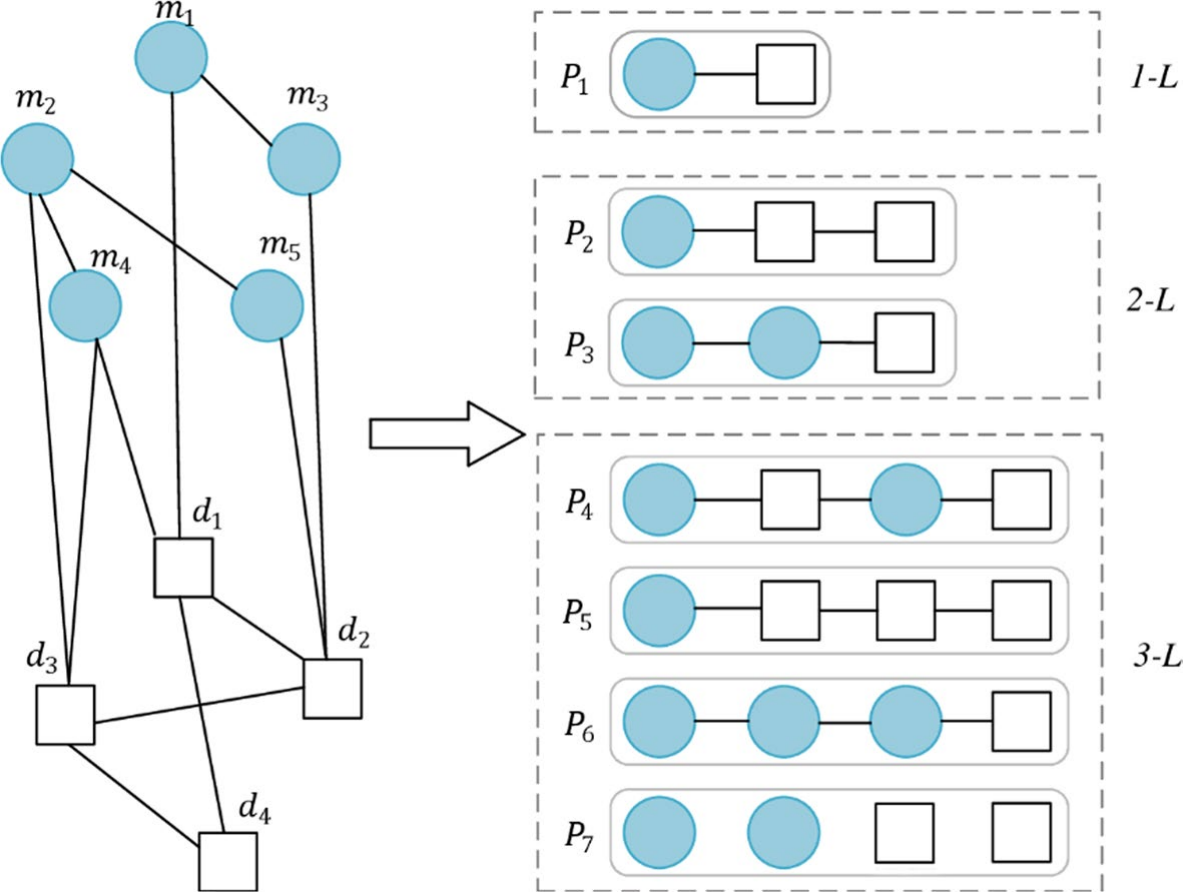
Example of meta-paths with different Length

Firstly, we define that meta-path $P$ with *L-Length* as a sequence is in form of $m\to {N}_{1}\to \cdots {N}_{i}\to \cdots d$. Where, $m$ and $d$ is from the verified miRNA and disease pair with HMDD2.0, ${N}_{i}\in \left\{M,D\right\}$. Different types of meta-path can help understand the season why two nodes are closely related to each other. Because the paths from one node to another can also be associated with multiple types, which construct the different semantics of the paths. For example, a meta-path type of $D\to D\to {\rm{M}}$ shows that if a disease is associated with a miRNA, then other disease who is similar to the disease will be potential associated with the miRNA. A meta-path type of $D\to M\to {\rm{M}}$ shows that if a miRNA is associated with a disease, then other miRNA who is similar to the miRNA will be potential associated with the disease. There are different mete-path instances with *L-Length* between the identified *m* and *d* as shown in Fig. 10. For example, the confirmed ${m}_{2}$and ${d}_{2}$ pair have different instances with different length, one meta-path instance ${P}_{7}={m}_{2}\to {m}_{2}\to {d}_{3}\to {d}_{2}$ is a *3-Length* and ${P}_{2}={m}_{4}\to {d}_{1}\to {d}_{4}$ is a *2-Length*.

Finally, all meta-path instances of the confirmed miRNA and disease in network are extracted.

### Pair embedding of MiRNA-disease

#### Linear transformations of MiRNAs and diseases

We take the $i$-th row in the miRNA similarity matrix $SM$ as the initial features of the $i$-th miRNA. In the same way, we regard the *j*-th row in the disease similarity matrix $SD$ as the feature of the $j$-th disease. Then, the initial features of miRNAs and diseases projected into the same vector with linear transformations because of the difference of dimensions.

We project the feature of a miRNA $r$ into the $Z$-dimensional space as follows:

11 $${\varvec{h}}_{r} = {\varvec{W}}^{R} \cdot {\varvec{x}}_{r}$$

Similarly, the initial feature of disease *d* is projected into the $Z$-dimensional space as follows:

12 $${\varvec{h}}_{d} = {\varvec{W}}^{D} \cdot {\varvec{x}}_{d} { }$$

where ${{\varvec{h}}}_{r}, {{\varvec{h}}}_{d}$ is the projected feature of miRNA *r* and disease *d*, respectively. ${{\varvec{x}}}_{r}$ and ${{\varvec{x}}}_{d}$ are the initial feature of miRNA *r* and disease *d*. ${{\varvec{W}}}^{R}\in {\mathbb{R}}^{Z*m}$ is a linear transformation matrix to project the 495-dimensional matix into $Z$-dimensional space and ${{\varvec{W}}}^{D}\in {\mathbb{R}}^{Z*n}$ is a linear transformation matrix to project the 383-dimensional matix into $Z$-dimensional space.

In Fig. 9, the nodes with shadow are the transformed representation of the initial miRNA and disease.

#### MLP encoder of miRNA-disease interactions

Given a miRNA embedding ${{\varvec{h}}}_{r} \in {\mathbb{R}}^{Z}$ and a disease embedding ${{\varvec{h}}}_{d}\in {\mathbb{R}}^{Z}$ as $Com\left({{\varvec{h}}}_{{\varvec{r}}},{{\varvec{h}}}_{d}\right)\in {\mathbb{R}}^{4Z}$, we use a $m$-layer multi-layer perceptron (MLP) to embed miRNA-disease interaction (${{\varvec{h}}}_{r},{{\varvec{h}}}_{d}$) into *Z*-dimensional vector. The pair embedder is ${\varvec{g}}\left(r,d\right)$. Firstly, miRNA embedding and disease embedding is combined to form the initial input of MLP.

13 $${\varvec{h}}^{\left( 0 \right)} = {\varvec{Com}}\left( {{\varvec{h}}_{r} ,{\varvec{h}}_{d} } \right) = \left[ {{\varvec{h}}_{r} ;{\varvec{h}}_{d} ;{\varvec{h}}_{r} \circ {\varvec{h}}_{d} ;{\varvec{h}}_{r} + {\varvec{h}}_{d} } \right]$$

14 $${\varvec{h}}^{{\left( {\varvec{l}} \right)}} = \left\{ {\begin{array}{*{20}l} {ReLU\left( {{\varvec{W}}^{{\left( {\varvec{l}} \right)}} \cdot {\varvec{h}}^{{\left( {{\varvec{l}} - 1} \right)}} + {\varvec{b}}^{{\left( {\varvec{l}} \right)}} } \right),\quad 0 < l < m} \hfill \\ {{\varvec{W}}^{{\left( {\varvec{l}} \right)}} \cdot {\varvec{h}}^{{\left( {{\varvec{l}} - 1} \right)}} + {\varvec{b}}^{{\left( {\varvec{l}} \right)}} ,\quad l = m} \hfill \\ \end{array} \varvec{ }} \right.$$

15 $${\varvec{g}}\left( {{\varvec{r}},{\varvec{d}}} \right) = {\varvec{h}}^{{\left( {\varvec{m}} \right)}}$$

where ° denotes element-wise vector multiplication, $ReLU$($x$) denotes $max\left(0,x\right)$ and ${\varvec{g}}\left({\varvec{r}},{\varvec{d}}\right)\in {\mathbb{R}}^{X}$. We employ dropout on the hidden layers and regarded the last layer output of MLP as the pair embedding. We take ***g***(·) as a 2-layered MLP, which each layer has 100 hidden units.

#### Validity of pair embedding

Recall that one of the limitations of node embedding is that it inadvertently makes a miRNA and a disease similar to each other if they frequently appear together within the meta-path, whether or not the miRNA is associated with disease. Then, we present a pair validity classifier $\pi$: ${\mathbb{R}}^{X}\to {\mathbb{R}}$ to discriminate whether the miRNA-disease pair is a valid pair or not, which is formulated by binary cross-entropy loss as follows:

16 $$Loss_{N} = y_{r,d} \sigma \left[ {\pi \left( {{\varvec{g}}\left( {r,d} \right)} \right)} \right] + \left( {1 - y_{r,d} } \right)\left( {1 - \sigma \left[ {\pi \left( {{\varvec{g}}\left( {r,d} \right)} \right)} \right]} \right)$$

17 $$y_{r,d} = \left\{ {\begin{array}{*{20}l} {{ }1,} \hfill & {miRNA{ }r\;is\;associated\;with\;disease\;d} \hfill \\ {0,} \hfill & {{ }miRNA{ }r\;is\;not\;associated\;with\;disease\;d} \hfill \\ \end{array} } \right.$$

$\pi (\cdot )$ is a 2-layered MLP with ReLU activation.

### Meta-path based node embedding

#### Multi-head attention embedding of meta-path

Meta-paths are linked by a series of nodes, which can be employed to preserve the important structure information in heterogeneous networks. According to a meta-path instance $p$ connecting the confirmed miRNA $r$ with disease $d$, the measurable features of the connection are implied in the sequences of $p$. The sequence of $p$ is represented as ${\{ {\varvec{X}}}_{1},{{\varvec{X}}}_{2},\cdots {{\varvec{X}}}_{n-1},{{\varvec{X}}}_{n}\}$, where, ${{\varvec{X}}}_{1}={{\varvec{h}}}_{r}$, ${{\varvec{X}}}_{n}={{\varvec{h}}}_{d}$. Considering that different nodes in the meta path have different importance to the meta path, GRU can learn important nodes with the contributions to the sequence, which is suitable for sequential data learning. We use a GRU to generate a $Z$-dimensional vector for $p$. GRU calculates the hidden state ${{\varvec{h}}}_{t}$ with ${{\varvec{h}}}_{t-1}$ and ${{\varvec{X}}}_{t}$ as input, $t\in [1,n]$, which is shown as follows.

18 $${\varvec{z}}_{{\varvec{t}}} = \sigma \left( {{\varvec{W}}_{{{\varvec{zx}}}} \cdot{\varvec{X}}_{{\varvec{t}}} + {\varvec{W}}_{{{\varvec{zh}}}} \cdot {\varvec{h}}_{{{\varvec{t}} - 1}} + {\varvec{b}}_{{\varvec{z}}} } \right)$$

19 $${\varvec{r}}_{{\varvec{t}}} = \sigma \left( {{\varvec{W}}_{{{\varvec{rx}}}} \cdot {\varvec{X}}_{{\varvec{t}}} + {\varvec{W}}_{{{\varvec{rh}}}} \cdot {\varvec{h}}_{{{\varvec{t}} - 1}} + {\varvec{b}}_{{\varvec{r}}} } \right)$$

20 $${\varvec{g}}_{{\varvec{t}}} = tanh\left[ {{\varvec{W}}_{{{\varvec{hx}}}} \cdot {\varvec{X}}_{{\varvec{t}}} + {\varvec{W}}_{{{\varvec{hh}}}} \cdot \left( {{\varvec{r}}_{{\varvec{t}}} \circ {\varvec{h}}_{{{\varvec{t}} - 1}} } \right) + {\varvec{b}}_{{\varvec{h}}} } \right]$$

21 $${\varvec{h}}_{{\varvec{t}}} = {\varvec{z}}_{{\varvec{t}}} \circ {\varvec{h}}_{{{\varvec{t}} - 1}} + \left( {1 - {\varvec{z}}_{{\varvec{t}}} } \right) \circ {\varvec{g}}_{{\varvec{t}}}$$

where ${\varvec{\sigma}}$ is a sigmoid function, and ${{\varvec{W}}}_{{\varvec{z}}{\varvec{x}}}{\in {\mathbb{R}}}^{X\times Z}$, ${{\varvec{W}}}_{{\varvec{r}}{\varvec{x}}}{\in {\mathbb{R}}}^{X\times Z}$, ${{\varvec{W}}}_{{\varvec{h}}{\varvec{x}}}{\in {\mathbb{R}}}^{X\times Z}$, ${{\varvec{W}}}_{{\varvec{z}}{\varvec{h}}}\in {\mathbb{R}}^{X\times X}$, ${{\varvec{W}}}_{{\varvec{r}}{\varvec{h}}}\in {\mathbb{R}}^{X\times X}$, ${{\varvec{W}}}_{{\varvec{h}}{\varvec{h}}}\in {\mathbb{R}}^{X\times X}$, ${{\varvec{b}}}_{{\varvec{z}}}\in {\mathbb{R}}^{X},{{\varvec{b}}}_{{\varvec{r}}}\in {\mathbb{R}}^{X},{{\varvec{b}}}_{{\varvec{h}}}\in {\mathbb{R}}^{X}$.

We apply dropout to the hidden state update vector as *g*_*t*_ follows:

22 $${\varvec{h}}_{{\varvec{t}}} = {\varvec{z}}_{{\varvec{t}}} \circ {\varvec{h}}_{{{\varvec{t}} - 1}} + \left( {1 - {\varvec{z}}_{{\varvec{t}}} } \right) \circ \left( {{\varvec{d}}\left( {{\varvec{g}}_{{\varvec{t}}} } \right)} \right)$$

where ${\varvec{d}}(\cdot )$ is the dropout function defined as follows:

23 $${\varvec{d}}\left( {\varvec{X}} \right) = \left\{ {\begin{array}{*{20}l} {{\varvec{mask}} \circ {\varvec{X}}} \hfill & {if\;train\;phase} \hfill \\ {\left( {1 - q} \right){\varvec{X}}} \hfill & {otherwise} \hfill \\ \end{array} } \right.$$

where $q$ is the dropout rate and ${\varvec{m}}{\varvec{a}}{\varvec{s}}{\varvec{k}}$ is a vector, which is got from sampling from the Bernoulli distribution with success probability $1-q$.

We obtain an embedding matrix ***h***$\in {\mathbb{R}}^{n\times Z}$ after GRU training of meta-path instance $p$. $Z$-dimesnional vector is extracted by aggregating ${\varvec{h}}$ with attentive pooling. The contribution of each node in the meta-path instances is measured as follows:

24 $${\varvec{\alpha}}_{{\varvec{i}}} = \frac{{exp\left( {{\varvec{M}} \cdot {\varvec{h}}_{{\varvec{i}}} } \right)}}{{\mathop \sum \nolimits_{{{\varvec{j}} = 1}}^{{\varvec{n}}} exp\left( {{\varvec{M}} \cdot {\varvec{h}}_{{\varvec{j}}} } \right)}}$$

where ${\varvec{M}}\in {\mathbb{R}}^{Z}$is a trained attention parameter vector, $i\in \left[1,n\right],j\in [1,n]$.

The extracted vector is formed by a weighted sum of the vectors from the matrix ${\varvec{h}}$ as follows:

25 $${\varvec{h}}_{{{\varvec{r}},{\varvec{d}}}}^{{\varvec{p}}} = \mathop \sum \limits_{{{\varvec{i}} = 1}}^{{\varvec{n}}} {\varvec{\alpha}}_{{\varvec{i}}} \cdot {\varvec{h}}_{{\varvec{i }}}$$

To make the learning of attention parameter stable, we extend attention mechanism to multi-head attention, conduct attention *K* times independently and average their outputs as follows:

26 $${\varvec{h}}_{{{\varvec{r}},{\varvec{d}}}}^{{\varvec{p}}} = \frac{1}{K}\left( {\mathop \sum \limits_{{{\varvec{k}} = 1}}^{{\varvec{K}}} \mathop \sum \limits_{{{\varvec{i}} = 1}}^{{\varvec{n}}} {\varvec{\alpha}}_{{\varvec{i}}}^{{\varvec{k}}} \cdot {\varvec{h}}_{{\varvec{i}}} } \right)$$

where ΣΣ indicates concatenation, ${\varvec{\alpha }}_{{\varvec{i}}}^{{\varvec{k}}}$are normalized attention coefficients in the $K$-th attention.

#### Attention-aware fusion of multiple meta-path instances to represent miRNA-disease associations

For meta-path instances connecting the confirmed miRNA $r$ and disease $d$, the meta-path instances may have different length. The meta-path instances with the same meta-path length exhibit diverse contributions to the connection between *r*_*i*_ and *d*_*j*_ as the difference of nodes in the sequences, which we call meta-path type. For example, ${m}_{2}\to {m}_{4}\to {d}_{3}\to {d}_{4}$ and $m\to {m}_{4}\to {d}_{1}\to {d}_{4}$ are listed in Fig. 10. Since the related information involved in two meta-path instances are not the same. To merge the global information of different meta-path instances with the same length to indicate the connection between $r$ and *d*, we joint into an attention.

27 $${\varvec{e}}^{{\varvec{p}}} = ReLU\left( {{\varvec{att}}_{p} \cdot {\varvec{h}}_{{{\varvec{r}},{\varvec{d}}}}^{{\varvec{p}}} } \right)$$

28 $$\left( {\varvec{e^{\prime}}} \right)^{p} = \frac{{\exp \left( {{\varvec{e}}^{{\varvec{p}}} } \right)}}{{\mathop \sum \nolimits_{q \in P} \exp \left( {{\varvec{e}}^{{\varvec{q}}} } \right)}}$$

29 $${\varvec{h}}_{{{\varvec{r}},{\varvec{d}}}}^{{\varvec{P}}} = sigmoid\left( {\mathop \sum \limits_{p \in P} \left( {\varvec{e^{\prime}}} \right)^{{\varvec{p}}} \cdot {\varvec{h}}_{{{\varvec{r}},{\varvec{d}}}}^{{\varvec{p}}} } \right)$$

where ${{{\varvec{a}}{\varvec{t}}{\varvec{t}}}_{{\varvec{p}}}\in {\mathbb{R}}}^{Z}$ is the parameter in meta-path instance $p$. ${{\varvec{e}}}^{p}$ indicates the contribution of meta-path instance $p$ of ${r}_{i}$ and ${d}_{j}$. ${({{\varvec{e}}}^{{^{\prime}}})}^{{\varvec{p}}}$ is normalized with the softmax function among all meta-path instances with meta-path type $P$. For all $p\in P$, the comprehensive representation the connection between ${r}_{i}$ and ${d}_{j}$ can be obtained by the weighted sum of all meta-path instances as shown in Formula (29).

#### Attention-aware fusion of multiple meta-paths to represent miRNA-disease associations

We define meta-path type as ${P}_{i}$, $i\in \left[1,N\right]$ and the features of the confirmed miRNA ${r}_{i}$ and disease ${d}_{j}$ association by different meta-path type as ${{\varvec{h}}}^{{P}_{i}}{\in {\mathbb{R}}}^{Z}$. Supposing the different contributions of different types and length, attention mechanisms are employed to obtain the ultimate representation.

30 $${\varvec{w}}^{{P_{i} }} = ReLU\left( {{\varvec{att}}_{{P_{i} }} \cdot {\varvec{h}}_{{{\varvec{r}},{\varvec{d}}}}^{{P_{i} }} } \right)$$

31 $${\varvec{w}}^{{\prime}{P_{i} }} = \frac{{\exp \left( {{\varvec{w}}^{{{\varvec{P}}_{{\varvec{i}}} }} } \right)}}{{\mathop \sum \nolimits_{{P_{i} \in P}} \exp \left( {{\varvec{w}}^{{{\varvec{P}}_{{\varvec{i}}} }} } \right)}}$$

32 $${\varvec{h}}_{{{\varvec{r}},{\varvec{d}}}}^{{\varvec{P}}} = \mathop \sum \limits_{{P_{i} \in P}} {\varvec{w}}{^{\prime}}^{{P_{i} }} \cdot {\varvec{h}}_{{{\varvec{r}},{\varvec{d}}}}^{{P_{i} }}$$

where ${{\varvec{a}}{\varvec{t}}{\varvec{t}}}_{{P}_{i}}{\in {\mathbb{R}}}^{Z}$ is the parameter with different path length ${P}_{i}$. ${{\varvec{w}}}^{{P}_{i}}$ indicates that the contribution of meta-path type ${P}_{i}$ to the connection. ${{\varvec{w}}{^{\prime}}}^{{P}_{i}}$is normalized with the softmax function of all the meta-paths. So, ${{\varvec{h}}}_{r,d}^{p}{\in {\mathbb{R}}}^{Z}$ represents all math-path with path length attention.

Finally, the representations of miRNA $r$ and disease $d$ interactions with significant information of meta-paths are modeled by the above-mentioned mechanisms.

#### Predicting MiRNA-disease associations with combined embedding

Finally, we get the ultimate representation of miRNA and disease ${{\varvec{h}}}_{u}^{P}$, including the total information of miRNA and disease associations. The parameters of ${{\varvec{W}}}^{R}{,{\varvec{W}}}^{D}{,{\varvec{a}}{\varvec{t}}{\varvec{t}}}_{p}$ and ${{\varvec{a}}{\varvec{t}}{\varvec{t}}}_{pi}$ are trained in order to gain features as correct as possible. The primary purpose for training our model is to make distance between two nodes who are related in miRNA and disease heterogeneous network as small as possible. Meanwhile, we want to make pair embedding and meta-path based node embedding similar. Thence, we predicting miRNA-disease associations with combined embedding.

We obtain the cross entropy for meta-path based node embedding as follows:

33 $$Loss_{M} = \mathop \sum \limits_{{\left( {r,d} \right) \in {\mathcal{P}}}} \log sigmoid\left( {{\varvec{h}}\left( {r,d} \right)} \right){ } - \mathop \sum \limits_{{\left( {r,d} \right) \notin {\mathcal{P}}}} \log sigmoid\left( { - {\varvec{h}}\left( {r,d} \right)} \right)$$

where $\mathcal{P}$ is the set of positive pairs with the supported relationships. The parameters can be learned by minimizing the following loss function. We combine the above two loss functions to gain the ultimate loss function as follows:

34 $$Loss = Loss_{N} + \lambda Loss_{M} - (1 - {\uplambda })Loss_{reg}$$

${Loss}_{Reg}$ is the regularization to prevent overfitting. We analyzed the AUC with the value of $\lambda$ from 0 to 1 with the interval of 0.1. It denotes that When $\lambda$ is set to 0.5, CEMDA achieved the better result. Thus, we set $\lambda$ to 0.5.

## Supplementary Information

**Additional file 1**. Supplementary tables for case studies.

## Data Availability

The datasets that support the findings of this study are available in https://github.com/liubailong/CEMDA. A web service for CEMDA is available at http://132.232.17.50:8080/CEMDA.jsp

## Abbreviations

CEMDA: Combined embedding model to predict MiRNA-disease associations
GRU: Gate recurrent unit
MLP: Multi-layer perceptron
LOOCV: Global leave-one-out cross validation
miRNAs: Microribonucleic acids
MeSH: Medical subject headings

## References

[1] Huang HY, Lin YCD, Li J, Huang KY, Shrestha S, Hong HC (2020). miRTarBase 2020 updates to the experimentally valida-ted microRNA-target interaction database. Nucleic Acids Res.

[2] Chen PP, Wang DD, Chen H, Zhou ZZ, He XL (2016). The non-essentiality of essential genes in yeast provides therapeutic insights into a human disease. Genome Res.

[3] Zheng Y, Jiang SB, Zhang HY, Zhang R, Gong DQ (2015). Detection of miR-33 expression and the verification of its target genes in the fatty liver of geese. Int J Mol Sci.

[4] Shefa U, Jung JY (2019). Comparative study of microarray and experimental data on Schwann cells in peripheral nerve degeneration and regeneration: big data analysis. Neural Regen Res.

[5] Chen X, Xie D, Zhao Q, You ZH (2019). MicroRNAs and complex diseases: from experimental results to computational models. Brief Bioinform.

[6] Zhang H, Liang Y, Han SY, Peng C, Li Y (2019). Long noncoding RNA and protein interactions: from experimental results to computational models based on network methods. Int J Mol Sci.

[7] Jiang Q, Wang G, Wang Y. An approach for prioritizing disease-related microRNAs based on genomic data integration. In: Proceedings of the international conference on biomedical engineering and informatics. 2010; 2270–4.

[8] Xuan P, Han K, Guo M, Gao YH, Li JB, Ding J (2013). Prediction of microRNAs associated with human diseases based on weighted k most similar neighbors. PLoS ONE.

[9] Chen M, Liao B, Li ZJ (2018). Global similarity method based on a two-tier random walk for the prediction of microRNA-disease association. Sci Rep.

[10] Chen X, Yan CC, Zhang X, You ZH, Deng LX, Liu Y (2016). WBSMDA: within and between score for MiRNA-disease association prediction. Sci Rep.

[11] Zhao HC, Kuang LN, Wang L (2018). Prediction of MicroRNA-disease associations based on distance correlation set. BMC Bioinform.

[12] Jiang Q, Wang G, Zhang T, et al. Predicting human microRNA-disease associations based on support vector machine. IEEE international conference on bioinformatics and biomedicine. 2010, pp. 467–472. 10.1504/ijdmb.2013.056078 24417022

[13] Chen X, Yan GY (2014). Semi-supervised learning for potential human microRNA-disease associations inference. Sci Rep.

[14] Liang C, Yu SP, Luo JW (2019). Adaptive multi-view multi-label learning for identifying disease-associated candidate miRNAs. PLoS Comput Biol.

[15] Chen X, Sun LG, Zhao Y. NCMCMDA: miRNA-disease association prediction through neighborhood constraint matrix completion. Briefings in Bioinformatics. 2020. 10.1093/bib/bbz159 31927572

[16] Zhao Y, Chen X, Yin J (2019). Adaptive boosting-based computational model for predicting potential miRNA-disease associations. Bioinformatics.

[17] Chen X, Wang CC, Yin J, You ZH (2018). Novel human miRNA-disease association inference based on random forest. Mol Ther Nucleic Acids.

[18] Chen X, Wang L, Qu J, Guan NN, Li JQ (2018). Predicting miRNA-disease association based on inductive matrix completion. Bioinformatics.

[19] Jiang YT, Liu BT, Yu LH, Yan CG, Bian HJ (2018). Predict MiRNA-disease association with collaborative filtering. Neuroinformatics.

[20] Mao G, Wang SL, Zhang W (2019). Prediction of potential associations between MicroRNA and disease based on bayesian probabilistic matrix factorization model. J Comput Biol.

[21] Chen ZH, Wang XK, Gao P, Liu HJ, Song BS (2019). Predicting disease related microRNA based on similarity and topology. Cells.

[22] Zeng XX, Wang W, Deng GS, Bing JX, Zou Q (2019). Prediction of potential disease-associated MicroRNAs by using neural networks. Mol Ther Nucleic Acids.

[23] Gong YC, Niu YQ, Zhang W, Li XH (2019). A network embedding-based multiple information integration method for the MiRNA-disease association prediction. BMC Bioinform.

[24] Zhang C, Chao Huang, Lu Yu, et al. Camel: content-aware and meta-path augmented metric learning for author identification. WWW. 2018

[25] Wang Y, Zheng FS, Wang ZB, Lu JB, Zhang HY (2020). Circular RNA circ-SLC7A6 acts as a tumor suppressor in non-small cell lung cancer through abundantly sponging miR-21. Cell Cycle.

[26] Zhang XJ, Li YL, Qi PF, Ma ZL (2018). Biology of MiR-17-92 cluster and its progress in lung cancer. Int J Med Sci.

[27] Sun Q, Hang M, Guo XD, Shao WL, Zeng GQ (2013). Expression and significance of miRNA-21 and BTG2 in lung cancer. Tumor Biol.

[28] Shi HB, Xu J, Zhang GD, Xu LD, Li CQ, Wang L (2013). Walking the interactome to identify human miRNA-disease associations through the functional link between miRNA targets and disease genes. BMC Syst Biol.

[29] Minh NT, Wu YH. Integrating meta-path similarity with user preference for top-N recommendation. In: International conference on technologies and applications of artificial intelligence (TAAI). 2019, pp. 1–6.

[30] Li Y, Qiu CX, Tu J, Geng B, Yang JC, Jiang TZ (2013). HMDD v2.0: a database for experimentally supported human microRNA and disease associations. Nucleic Acids Res.

[31] Li SR, Xie MZ, Liu XQ (2019). A novel approach based on bipartite network recommendation and KATZ model to predict potential micro-disease associations. Front Genet.

[32] Yang Z, Ren F, Liu CN, He SM, Sun G, Gao Q (2010). dbDEMC: a database of differentially expressed miRNAs in human cancers. BioMed Central.

[33] Ruepp A, Kowarsch A, Schmidl D, Buggenthin F, Brauner B, Dunger I (2010). PhenomiR: a knowledgebase for microRNA expression in diseases and biological processes. Genome Biol.

[34] Siegel RL, Miller KD, Jemal A (2017). CA: a cancer journal for clinicians. Cancer Stat.

[35] Xiao WD, Zhong YC, Wu LL, Yang DX, Ye SQ, Zhang M (2019). Prognostic value of microRNAs in lung cancer: a systematic review and meta-analysis. Mol Clin Oncol.

[36] Li YX, Cui XM, Li YD, Zhang TT, Li SY (2018). Upregulated expression of miR-421 is associated with poor prognosis in non-small-cell lung cancer. Cancer Manag Res.

[37] Mansoori B, Mohammadi A, Ghasabi M, Shirjang S, Dehghan R, Montazeri V (2019). MiR-142-3p as tumor suppressor miRNA in the regulation of tumorigenicity, invasion and migration of human breast cancer by targeting Bach-1 expression. J Cell Physiol.

[38] He YJ, Deng F, Zhao SJ, Zhong SL, Zhao JH, Wang DD (2019). Analysis of miRNA–mRNA network reveals miR-140-5p as a suppressor of breast cancer glycolysis via targeting GLUT1. Epigenomics.

[39] Voss G, Haflidadóttir BS, Järemo H, Persson M, Ivkovic CT, Wikström P, Ceder Y (2019). Regulation of cell–cell adhesion in prostate cancer cells by microRNA-96 through upregulation of E-Cadherin and EpCAM. Carcinogenesis.

[40] Huang Z, Shi JC, Gao YX, Cui CM, Zhang S, Li JW (2018). HMDD v3.0: a database for experimentally supported human microRNA-disease associations. Nucleic Acids Res.

[41] Wang D, Wang J, Lu M, Song F, Cui QH (2010). Inferring the human microRNA functional similarity and functional network based on microRNA associated diseases. Bioinformatics.

[42] Lipscomb CE (2000). Medical subject headings (MeSH). Bull Med Lib Assoc.

