# Supplementary material for: Combined embedding model for MiRNA-disease association prediction
Source: BMC Bioinformatics. 2021 Mar 25;22:161. doi: 10.1186/s12859-021-04092-w (PMC7995599; doi:10.1186/s12859-021-04092-w)
Supplement: Supplementary file 1 — Additional file 1. Supplementary tables for case studies. [file 12859_2021_4092_MOESM1_ESM.docx]

**Supplementary Tables for Case Studies**

Table S1. Top 50 miRNAs associated with prostate cancers

| miRNA | Evidence | miRNA | Evidence |
| --- | --- | --- | --- |
| hsa-mir-16 | dbDEMC, PhenomiR | hsa-mir-23b | dbDEMC, PhenomiR |
| hsa-mir-197 | dbDEMC, PhenomiR | hsa-mir-92 | dbDEMC, PhenomiR |
| hsa-mir-181b | dbDEMC, PhenomiR | hsa-mir-302d | PhenomiR |
| hsa-mir-23b | dbDEMC, PhenomiR | hsa-mir-195 | dbDEMC, PhenomiR |
| hsa-mir-101 | dbDEMC, PhenomiR | hsa-mir-130b | dbDEMC, PhenomiR |
| hsa-mir-26a | dbDEMC, PhenomiR | hsa-let-7i | dbDEMC, PhenomiR |
| hsa-mir-17 | dbDEMC, PhenomiR | hsa-let-7c | dbDEMC, PhenomiR |
| hsa-mir-146a | dbDEMC, PhenomiR | hsa-mir-92a | dbDEMC, PhenomiR |
| hsa-mir-182 | dbDEMC, PhenomiR | hsa-mir-184 | dbDEMC, PhenomiR |
| hsa-mir-122 | dbDEMC, PhenomiR | hsa-mir-130a | dbDEMC, PhenomiR |
| hsa-mir-93 | dbDEMC, PhenomiR | hsa-mir-155 | dbDEMC, PhenomiR |
| hsa-mir-10b | dbDEMC, PhenomiR | hsa-mir-20b | dbDEMC |
| hsa-mir-31 | dbDEMC, PhenomiR | hsa-mir-29a | dbDEMC, PhenomiR |
| hsa-let-7g | dbDEMC, PhenomiR | hsa-mir-191 | dbDEMC, PhenomiR |
| hsa-mir-181d | dbDEMC, PhenomiR | hsa-mir-137 | dbDEMC, PhenomiR |
| hsa-mir-297 | dbDEMC, PhenomiR | hsa-mir-10a | dbDEMC, PhenomiR |
| hsa-mir-23a | dbDEMC, PhenomiR | hsa-mir-92b | dbDEMC |
| hsa-mir-27a | dbDEMC, PhenomiR | hsa-mir-186 | dbDEMC, PhenomiR |
| hsa-mir-33b | dbDEMC, PhenomiR | hsa-mir-194 | dbDEMC, PhenomiR |
| hsa-mir-19a | dbDEMC, PhenomiR | hsa-mir-15a | dbDEMC, PhenomiR |
| hsa-mir-1 | dbDEMC, PhenomiR | hsa-mir-26b | dbDEMC, PhenomiR |
| hsa-mir-27b | dbDEMC, PhenomiR | hsa-let-7d | dbDEMC, PhenomiR |
| hsa-mir-218 | dbDEMC, PhenomiR | hsa-mir-20a | dbDEMC, PhenomiR |
| hsa-let-7e | dbDEMC, PhenomiR | hsa-mir-301a | dbDEMC, PhenomiR |
| hsa-mir-373 | dbDEMC, PhenomiR | hsa-mir-363 | dbDEMC |

Table S2. The top 50 miRNAs associated with pancreatic cancers

| miRNA | Evidence | miRNA | Evidence |
| --- | --- | --- | --- |
| hsa-mir-23b | dbDEMC, PhenomiR | hsa-mir-200 | dbDEMC, PhenomiR |
| hsa-mir-99b | dbDEMC, PhenomiR | hsa-mir-130b | dbDEMC, PhenomiR |
| hsa-mir-136 | dbDEMC, PhenomiR | hsa-mir-1 | dbDEMC, PhenomiR |
| hsa-mir-9 | dbDEMC, PhenomiR | hsa-mir-140 | dbDEMC, PhenomiR |
| hsa-mir-28 | dbDEMC, PhenomiR | hsa-mir-149 | dbDEMC, PhenomiR |
| hsa-mir-30d | dbDEMC, PhenomiR | hsa-mir-22 | dbDEMC, PhenomiR |
| hsa-mir-29a | dbDEMC, PhenomiR | hsa-mir-27b | dbDEMC, PhenomiR |
| hsa-mir-33b | dbDEMC, PhenomiR | hsa-mir-30 | dbDEMC, PhenomiR |
| hsa-mir-189 | dbDEMC | hsa-mir-93 | dbDEMC, PhenomiR |
| hsa-mir-193a | dbDEMC, PhenomiR | hsa-mir-18b | dbDEMC, PhenomiR |
| hsa-mir-151 | dbDEMC, PhenomiR | hsa-mir-134 | dbDEMC, PhenomiR |
| hsa-mir-144 | dbDEMC, PhenomiR | hsa-mir-135a | dbDEMC, PhenomiR |
| hsa-mir-127 | dbDEMC, PhenomiR | hsa-mir-184 | dbDEMC, PhenomiR |
| hsa-let-7 | dbDEMC, PhenomiR | hsa-mir-29c | dbDEMC, PhenomiR |
| hsa-mir-26 | dbDEMC, PhenomiR | hsa-mir-20b | dbDEMC, PhenomiR |
| hsa-mir-19b | dbDEMC, PhenomiR | hsa-mir-138 | dbDEMC, PhenomiR |
| hsa-mir-129 | dbDEMC, PhenomiR | hsa-mir-152 | dbDEMC, PhenomiR |
| hsa-mir-130a | dbDEMC, PhenomiR | hsa-mir-133a | dbDEMC, PhenomiR |
| hsa-mir-19a | dbDEMC, PhenomiR | hsa-mir-202 | dbDEMC |
| hsa-mir-208b | dbDEMC | hsa-mir-154 | dbDEMC, PhenomiR |
| hsa-mir-148b | dbDEMC, PhenomiR | hsa-mir-33a | dbDEMC, PhenomiR |
| hsa-mir-30e | dbDEMC, PhenomiR | hsa-mir-181c | PhenomiR |
| hsa-mir-125a | dbDEMC, PhenomiR | hsa-mir-92 | dbDEMC, PhenomiR |
| hsa-mir-215 | dbDEMC, PhenomiR | hsa-mir-98 | dbDEMC, PhenomiR |
| hsa-mir-320c | dbDEMC | hsa-mir-185 | dbDEMC, PhenomiR |

Table S3. The top 50 miRNAs associated with colorectal cancers

| miRNA | Evidence | miRNA | Evidence |
| --- | --- | --- | --- |
| hsa-let-7c | dbDEMC, PhenomiR, HMDD | hsa-mir-374b | dbDEMC, PhenomiR, HMDD |
| hsa-mir-22 | dbDEMC, PhenomiR, HMDD | hsa-mir-100 | dbDEMC, PhenomiR, HMDD |
| hsa-mir-124a | dbDEMC, HMDD | hsa-mir-33b | dbDEMC, HMDD |
| hsa-let-7g | dbDEMC, PhenomiR, HMDD | hsa-mir-153 | dbDEMC, PhenomiR, HMDD |
| hsa-mir-99b | dbDEMC, PhenomiR, HMDD | hsa-mir-29a | dbDEMC, PhenomiR, HMDD |
| hsa-mir-21 | dbDEMC, PhenomiR, HMDD | hsa-mir-27b | dbDEMC, PhenomiR, HMDD |
| hsa-mir-128 | dbDEMC, PhenomiR, HMDD | hsa-mir-95 | dbDEMC, PhenomiR, HMDD |
| hsa-mir-206 | dbDEMC, HMDD | hsa-mir-154 | dbDEMC, HMDD |
| hsa-mir-33a | dbDEMC, PhenomiR, HMDD | hsa-mir-210 | dbDEMC, PhenomiR, HMDD |
| hsa-mir-18b | dbDEMC,HMDD | hsa-mir-214 | dbDEMC, PhenomiR, HMDD |
| hsa-mir-32 | dbDEMC, PhenomiR, HMDD | hsa-mir-223 | dbDEMC, PhenomiR, HMDD |
| hsa-mir-190b | dbDEMC | hsa-mir-34b | dbDEMC, PhenomiR, HMDD |
| hsa-mir-122 | dbDEMC, PhenomiR, HMDD | hsa-mir-29c | dbDEMC, PhenomiR, HMDD |
| hsa-mir-26a | dbDEMC, PhenomiR, HMDD | hsa-mir-106a | dbDEMC, PhenomiR, HMDD |
| hsa-mir-34a | dbDEMC, PhenomiR, HMDD | hsa-mir-34a | dbDEMC, PhenomiR, HMDD |
| hsa-let-7f | dbDEMC, PhenomiR | hsa-mir-145 | dbDEMC, PhenomiR |
| hsa-mir-15b | dbDEMC, PhenomiR, HMDD | hsa-mir-99 | PhenomiR |
| hsa-mir-28 | dbDEMC, PhenomiR, HMDD | hsa-mir-320c | dbDEMC |
| hsa-mir-101 | dbDEMC, PhenomiR, HMDD | hsa-mir-136 | dbDEMC |
| hsa-mir-137 | dbDEMC, PhenomiR, HMDD | hsa-mir-92 | dbDEMC, PhenomiR |
| hsa-mir-184 | dbDEMC, PhenomiR, HMDD | hsa-mir-23b | dbDEMC, PhenomiR, HMDD |
| hsa-mir-19a | dbDEMC, PhenomiR, HMDD | hsa-mir-20b | dbDEMC, HMDD |
| hsa-mir-130b | dbDEMC, PhenomiR, HMDD | hsa-mir-29b | dbDEMC, PhenomiR, HMDD |
| hsa-mir-133b | dbDEMC, PhenomiR, HMDD | hsa-mir-203 | dbDEMC, PhenomiR, HMDD |
| hsa-mir-214 | dbDEMC, PhenomiR, HMDD | hsa-mir-193b | dbDEMC, HMDD |
